# Valorization of spent black tea by recovery of antioxidant polyphenolic compounds: Subcritical solvent extraction and microencapsulation

**DOI:** 10.1002/fsn3.1726

**Published:** 2020-06-28

**Authors:** D.S.W. Rajapaksha, Naoto Shimizu

**Affiliations:** ^1^ Laboratory of Agricultural Bio‐system Engineering Graduate School of Agriculture Hokkaido University Hokkaido Japan; ^2^ Research Faculty of Agriculture / Field Science Center for Northern Biosphere Hokkaido University Hokkaido Japan

**Keywords:** antioxidant phenolic compounds, microencapsulation, response surface methodology, spent black tea, subcritical solvent extraction

## Abstract

Spent black tea (SBT), waste remaining after producing tea beverages, is potentially an underutilized source of antioxidant phenolic compounds. This study evaluated the integrated processes of subcritical solvent extraction of polyphenols from SBT followed by microencapsulation to improve the stability of obtained extract. Optimization of extraction conditions was carried out by response surface methodology for the best recovery of antioxidant phenolic compounds. Two variables [temperature (°C) and ethanol concentration (%)] were used to design the optimization model using central composite inscribed. Extraction temperature of 180°C and ethanol concentration of 71% were optimal for the highest yield of total polyphenols (126.89 mg gallic acid equiv./g SBT) and 2,2‐diphenyl‐1‐picrylhydrazyl scavenging activity (69.08 mg gallic acid equiv./g SBT). The extract was encapsulated using pectin, sodium caseinate, and a blend of these compounds (ratio 1:1) as wall materials by spray drying. The wall material significantly influenced (*p* < .05) encapsulation efficiency, particle size, morphology, thermal stability, crystallinity, and storage stability. The blend of wall materials produced an amorphous powder with the highest phenolic retention (94.28%) in the accelerated storage at 45°C for 40 days. The microcapsules prepared with sodium caseinate were smaller with lowest mean diameter and highest thermal stability than the other types of materials. Obtained microencapsulates have potential use in different food systems to enhance their antioxidant property.

## Graphical abstract

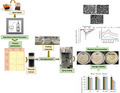

## 1 INTRODUCTION

Besides water, black tea is the most consumed beverage in the world. It is produced by a process that consists of withering, rolling, fermenting, and drying the leaves of *Camellia synensis*. During the fermentation process, the tea leaves oxidize to produce multimeric polyphenols with high antioxidant potency (Łuczaj & Skrzydlewska, 2005). Of the different varieties of tea produced worldwide, green, black, oolong, yellow, white, and dark, 78% consists of black tea (Kosińska & Andlauer, 2014; Weerawatanakorn et al., 2015).

Because of its convenience of consumption, ready‐to‐drink (RTD) or bottled tea is produced commercially in most parts of the world, in particular, Japan. As a consequence, a large amount of spent black tea (SBT), the residues after manufacturing this tea product, is generated annually (Kondo, Hirano, Kita, Jayanegara, & Yokota, 2018). These used tea leaves mostly become waste with only a small percentage used as feedstock or turned to compost (Sagar, Pareek, Sharma, Yahia, & Lobo, 2018). As the conditions used for tea brewing are mild, a significant amount of polyphenols with a high antioxidant power is retained in SBT (Abdeltaif, SirElkhatim, & Hassan, 2018). Hence, utilizing food manufacturing waste such as SBT is a sustainable and economically attractive way to recover antioxidant phenolic compounds but an efficient method for extracting polyphenols from SBT has not yet been developed and tested.

Several techniques have been investigated for extracting phytochemicals from food waste (Sagar et al., 2018; Yanagida, Shimizu, & Kimura, 2005). Of these techniques, subcritical solvent extraction (SSE) is a greener and faster method (Munir, Kheirkhah, Baroutian, Quek, & Young, 2018), which uses a pressurized liquid kept below its critical point (374°C for water) and above its boiling point (100°C for water). These conditions allow fluids to remain in a liquid state due to the applied pressure and it creates low polar water with equivalent to organic solvents at ambient temperature (Shimizu, Ushiyama, & Itoh, 2019; Zhang, Baroutian, Munir, & Young, 2017; Zhang & Wolf 2019). This technique facilitates rapid extraction without the loss or changing the chemical integrity of thermolabile compounds (Essien, Young, & Baroutian, 2020). Combining subcritical water with an organic solvent such as ethanol and methanol has also been used to improve the yield, extraction time and solubility of compounds (Kwon & Chung, 2015; Pronyk & Mazza, 2009). Response surface methodology (RSM) is commonly used for optimizing the process parameters for the extraction of phytochemicals. This is a useful mathematical and statistical tool for defining the effect of independent variables and their interactions on a particular response, such as the yield.

However, effectiveness of polyphenols mainly depends on their stability, bioactivity, and bioavailability. The unsaturated bonds in the molecular structure of polyphenols make them vulnerable to oxidants, light, and heat, thus reducing their activity (Kailaspathy, 2015). Therefore, protecting phenolic compounds by encapsulation following their extraction would be a better way to maintain the structural integrity of polyphenols until their industrial application. The microencapsulation of phenolic extracts not only preserves them but also produces a powdered product that is convenient for food application. At present, spray drying is the most widely used technique for the microencapsulation of polyphenols and other heat labile compounds because of its short thermal contact time, cost‐effectiveness, and suitability for industrial application.

As well as the technique used for microencapsulation, selecting a coating or wall material is also crucial for efficient spray drying (Ushiyama & Shimizu, 2018). Of the different types of wall material, polysaccharides and protein agents are commonly used either alone or in combination because of their distinct properties. Pectin is a polysaccharide with strong film‐forming, gelling, and binding abilities. Its ability to form stable dispersions at low concentrations facilitates microencapsulation by spray drying (Rehman et al., 2019). Sodium caseinate is the salt of casein, a major milk protein fraction. Generally, milk proteins act as effective film‐formers and emulsifiers while polysaccharides act as filler materials (Augustin & Oliver, 2014).

Ultimately, encapsulated SBT would exhibit important properties that would facilitate the shelf life of polyphenols because the coating materials can act as a barrier against adverse environmental conditions.

Therefore, the objective of the present study is to develop a method for recovering phenolic compounds from SBT by integrating the processes of SSE optimization and the subsequent encapsulation of SBT using different wall materials by spray drying. The encapsulated powder using pectin, sodium caseinate, and a mixture of these compounds as wall materials will be characterized to evaluate their encapsulation efficiency, morphology and size, thermal stability, crystallinity, and storage stability.

## 2 MATERIALS AND CHEMICALS

Low grown unblended black tea was supplied by Nawa withana Kanda tea factory in Sri Lanka, gallic acid by Sigma‐Aldrich (Shanghai, China), 2,2‐diphenyl‐1‐picrylhydrazyl (DPPH) by Sigma‐Aldrich (Taufkirchen, Germany), Folin and Ciocalteu phenol reagent and casein sodium salt from bovine milk by Sigma‐Aldrich (St Louis, MO, USA), and pectin from citrus, sodium carbonate, ethanol, and methanol by Fujifilm Wako Pure Chemical Corp. (Osaka, Japan). Distilled water was used in all the experiments. All other chemicals and solvents used were analytical grade.

### 2.1 Preparation of spent black tea

Black tea leaves (20 g) were brewed in 1,000 ml of boiling water (100°C) for 6 min. The infusion was then filtered using a tea strainer, and the residue was dried in an air‐drying oven at 45°C overnight.

### 2.2 Subcritical solvent extraction

Subcritical solvent extraction was performed using an organic synthesizer (Chemi‐station PPV 3000, Tokyo Rikakikai Co. Ltd, Tokyo, Japan) with an agitator and an 11‐ml reactor with a maximum temperature and pressure of 200°C and 5 MPa, respectively. For each experimental run, 0.5 g of SBT was mixed with 10 ml of solvent at a solid: solvent ratio of 1 g: 20 ml. The extraction reactor was filled and then purged three times with nitrogen gas to remove the atmospheric oxygen present in the reactor vessel, and then, an initial pressure of 2.0 MPa was applied. The heating control was adjusted to obtain the desired temperature, which was then maintained for 10 min. During extraction, the agitation speed was kept at 17 *g* to prevent any local overheating and to increase the mass transfer. The extraction process was conducted in at various ethanol concentration (0%–100%) and temperature (100°C–180°C) ranges, based on the RSM design given in Table 1. After the extraction, the reactor was immediately cooled by placing it in a container of cold water. The extracts were filtered through filter paper (6 μm) under vacuum after the vessel pressure reached the initial pressure, and then, the filtrate was lyophilized. The lyophilized powder of SBT extract was stored at 4°C until further analysis.

**TABLE 1:** Coded levels for process variables used in the experimental design (central composite inscribed (CCI))

Independent variables	Coded levels				
	‐α (−1)	Low (−0.7)	Medium (0)	High (+0.7)	+α (+1)
Temperature °C	100	112	140	168	180
Ethanol concentration %	0	15	50	85	100

### 2.3 Determination of total phenolic content

The TPC of the dried extract was measured colorimetrically using the Folin–Ciocalteu (FC) method described by Dranca and Oroian (2016) with little modifications. Briefly, the dried extract obtained was diluted with a dilution factor of 100, and then, a 1.0‐ml aliquot of the extract in triplicate was transferred into a test tube and mixed thoroughly with 5.0 ml of FC reagent diluted 1:10 with distilled water. After keeping for 3 min, 5.0 ml of sodium carbonate (7.5%, w/v) was added and mixed. The mixtures were then allowed to stand for 1 hr in the dark before measuring the absorbance using a UV–Vis spectrophotometer **(**JASCO V‐560, JASCO corporation, Tokyo, Japan) at 756 nm against the blank. Gallic acid was used as the standard for preparation of the standard curve (7.812–250 μg/ml, *R* ^2^ = .998). The TPC values were expressed as milligrams of gallic acid equivalent/g (dry weight) material (mg GAE)/g SBT).

### 2.4 Determination of antioxidant activity

The scavenging capacity of SBT extract toward 2,2‐diphenyl‐1‐picrylhydrazyl free radicals (DPPH) was measured using a slightly modified method of Brand‐Williams, Cuvelier, and Berset (1995). For that purpose, 0.1 mM DPPH solution was prepared. DPPH reagent and properly diluted liquid extracts (dilution factor; 70) were mixed (2.9 ml + 0.1 ml) and incubated at room temperature for 20 min. Absorbance was further measured at 517 nm with UV–vis spectrophotometer **(**JASCO V‐560, JASCO corporation, Tokyo, Japan). Gallic acid was used as the standard for preparation of standard curve (3.90–62.5 μg/ml, *R* ^2^ = .997). DPPH scavenging capacity was expressed as milligrams of gallic acid equivalent/g (dry weight) material (mg GAE)/g SBT).

### 2.5 Response surface methodology design

The study was conducted as a two‐factor full factorial experiment with the influence of two independent variables (temperature and ethanol concentration) on the responses (total phenolic content and DPPH scavenging capacity) being evaluated (Table 2). The CCI design consisted of 13 experiments using 5 centers, 4 axial, and 4 factorial points. The experimental data obtained were fitted to a second order polynomial model of the form:

$$y=b_{o}+\sum_{i=1}^{n}\left(b_{i}x_{i}\right)+\sum_{i=1}^{n}(b_{\mathrm{ii}}x_{\mathrm{ii}}^{2})+\sum_{ij=1}^{n}\left(b_{\mathrm{ij}}x_{i}x_{j}\right),$$

where *y* is the predicted values of TPC or DPPH scavenging capacity; *x_i_*, the coded levels of the design variables (Temperature and ethanol concentration); *b_o_*, a constant; *b_i_*, the linear effect; *b_ii_*, the quadratic effect; and *b_ij_*, interaction effects.

**TABLE 2:** Central composite design – inscribed (CCI) matrix and observed results

Run	Process variables – real and (coded) values		Responses	
	Temperature (°C)	Ethanol concentration (%)	Extraction yield (mg GAE/g SBT)	
			TPC	DPPH activity
1	112 (−0.7)	15 (−0.7)	77.72	35.88
2	180 (+1)	50	124.43	58.78
3	100 (−1)	50 (0)	88.42	43.61
4	112 (−0.7)	85 (+0.7)	78.00	44.40
5	168 (+0.7)	85 (+0.7)	105.98	67.68
6	168 (+0.7)	15 (−0.7)	79.11	44.36
7	140 (0)	100 (+1)	61.36	45.87
8	140 (0)	0 (−1)	42.19	28.35
9	140 (0)	50 (0)	87.14	40.55
10	140 (0)	50 (0)	90.62	45.36
11	140 (0)	50 (0)	89.50	45.51
12	140 (0)	50 (0)	95.58	43.61
13	140 (0)	50 (0)	93.10	42.14

Abbreviations: DPPH, 2,2‐diphenyl‐1‐picrylhydrazyl radical scavenging ability; GAE, gallic acid equivalent; TPC, total phenolic content.

The statistical significance of differences between the mean values of variables was determined at the 5% probability level (*p* < .05), and the data were analyzed by ANOVA. Minitab 19.1.1 software (Minitab Inc., State College, PA, USA) was used to generate the surface plots and the optimized conditions. All assays for characterizing the SBT extract were performed in triplicate.

### 2.6 Process of encapsulation

#### 2.6.1 Preparation of feed solutions

The coating materials (3 g) were dissolved in 100 ml of distilled water at 90°C and then stirred until a clear dispersion was achieved. Three coating materials were evaluated as follows: 100% pectin (PE), 100% sodium caseinate (SCN), and a 50%:50% combination of pectin and sodium caseinate (PE + SCN). The prepared PE solution was kept at room temperature while the SCN and PE + SCN solutions were kept in a refrigerator overnight to allow complete hydration to occur.

The next day, SBT extract concentrated by a rotary evaporator (N‐1210 and SB‐1300 water bath, EYELA Tokyo Rikakikai Co., Ltd, Tokyo, Japan) was added dropwise to the prepared biopolymer solutions heated to 40°C with magnetic stirring at 21.5 *g* for 20 min. The prepared feed solutions were sonicated for 20 min then homogenized (HERACLES‐16g, Koike Precision Instruments, Tokyo, Japan) with stirring for 30 min before further processing. The feed solution contained 20 g of the carrier solution and 1 g of the concentrated SBT extract. All the prepared feed solutions were then spray dried.

#### 2.6.2 Measurement of viscosity

Before spray drying, the viscosity of all feed solutions was measured using a Sine‐wave Vibro Viscometer SV‐10 (A&D Co. Ltd., Tokyo, Japan). All measurements were carried out at room temperature. Each experiment was performed three times, and the average value was taken as the final value.

#### 2.6.3 Spray drying conditions

The liquid feeds were spray dried using a laboratory scale spray dryer OSK 55MO102 (Osaka Seimitsu Kikai Co. Ltd, Osaka, Japan). The values of the operational parameters established for the drying process were as follows: solid concentration, 3% (g/g); inlet air temperature, 140°C; outlet air temperature, 85°C ± 3°C; atomization pressure, 0.4 MPa; and feed flow rate, 5 ml/min. The spray nozzle diameter was 0.5 mm. The same conditions were used for all feed solution formulations, and each experiment was performed in duplicate. SBT extract with no added biopolymer coating material was spray dried under similar conditions to the other samples. The resulting powders were packed in zip‐lock bags covered with aluminum foil then stored in a refrigerator until further evaluation.

### 2.7 Characterization of powders

#### 2.7.1 Encapsulation efficiency

The encapsulation efficiency (EE%) of the powders was calculated as:

$$\text{EE}\%=\frac{\text{TPC}‐\text{SPC}}{\text{TPC}}\times 100,$$

where TPC is the total phenolic content, and SPC is the surface phenolic content (Kaderides & Goula, 2019).

TPC was determined by dissolving 10 mg of the sample in 4 ml of ethanol and methanol followed by thorough agitation and sonication for 40 min to completely break down the microencapsulates. Then, the solution was filtered through a 0.45‐μm filter. SPC was measured by washing a powder sample (10 mg) into a filter paper (0.45 μm) using 4 ml of ethanol and methanol.

#### 2.7.2 Morphology and particle size analyses

The morphology of the particles was examined using scanning electron microscopy (SEM, JSM‐6301F, JEOL Ltd., Tokyo, Japan) at a beam voltage of 10 kV and a working distance of 39 mm. From the micrographs, the particle diameter was calculated using ImageJ open‐source software (imagej.net). For measuring the size distribution, 100 particles were counted.

#### 2.7.3 Thermogravimetric analyses

Thermogravimetric analyses (TGA) were carried out using a Rigaku TG 8120 thermogravimetric analyzer (Rigaku Corp., Austin, TX, USA). Approximately 5 mg of the sample was placed in an aluminum pan with an empty pan used as a reference. The samples were heated from 25 to 600°C at 10°C/min under an argon atmosphere.

#### 2.7.4 Crystallinity of powders

The crystallinity of the encapsulated phenolic extract was evaluated using a Rigaku Rint‐Ultima III X‐ray diffractometer (Rigaku Corp.) with Cu‐Kα radiation generated at 40 kV/40 mA at a wavelength of 0.154187 nm. The scanning range was 5–40° 2θ at a speed of 2° 2θ/min. The degree of crystallinity was calculated as described by Ahmadian, Niazmand, and Pourfarzad (2019):

$$\text{CD}\;=\frac{I_{\text{net}}}{I_{\text{total}}}\times 100,$$

where CD is the degree of crystallinity (%); *I*_net_, the crystalline intensity of peaks; and *I*_total_, the overall intensity.

#### 2.7.5 Accelerated storage stability study

Glass vials containing three types of microencapsulated powders loaded with SBT extract (20 mg) were stored in an incubator at 45°C for 40 days. After given periods (0, 20, and 40 days), samples were collected from each batch, and then, their retained phenolic content was determined.

### 2.8 Statistical analysis

Statistical analyses were carried out using Minitab 19.1.1. (Minitab Inc., State College, PA, USA) to determine the significant differences (*p* < .05) between encapsulated samples, and one‐way analysis of variance (ANOVA) and Turkey′s multiple comparison test were used. The results were reported as the mean value of three repeated experimental data.

## 3 RESULTS AND DISCUSSION

### 3.1 Model fitting

The central composite inscribed design was used to optimize the factors (ethanol concentration and temperature) of SSE for extracting the polyphenols. Analysis of variance (ANOVA) showed a significant (*p* < .05) model *F* value with a nonsignificant lack of fit for all responses. The coefficient of determination (*R* ^2^) showed a good fit with the experimental data (.96 for TPC and .93 for DPHH activity) with little variation around the mean (Table 3). Therefore, it was assumed that the selected model could be used for optimizing the extraction of phenolics. The linear, quadratic, and interactive effects of ethanol concentration (%) and temperature (°C) significantly influenced (*p* < .05) the TPC and DPPH scavenging capacity of the SBT extracts. The regression coefficient (*β*) values of the identified variables were obtained by multiple linear regression (Table 3).

**TABLE 3:** Regression coefficients (β), coefficient of determination (R^2^), and *F* test value of the predicted second order polynomial models for TPC and DPPH activity

Regression coefficients		
	TPC	DPPH activity
Intercept		
*β*ₒ	266.1	141.2
Linear		
*β*_1_	−3.18^***^	−1.65^***^
*β*_2_	+.72^**^	−.14^***^
Quadratic		
*β*_11_	+.01^**^	+.01^**^
*β*_22_	−.01^***^	−.001^n.s.^
Interaction		
*β*_1_ *β*_2_	+.01^*^	+.003^*^
*R* ^2^	.96	.93
*F* value (model)	36.70	21.06
*F* value (lack of fit)	4.16	3.73

Note

Level of significance *p < .05, **p < .01, ***p < .001, n.s. nonsignificant at *p* > .05

Abbreviations: DPPH, 2,2‐diphenyl‐1‐picrylhydrazyl radical scavenging ability (mg GAE/g SBT); *R* ^2^, Coefficient of determination; TPC, total phenolic content (mg GAE/g SBT).

### 3.2 Effect of variables on total phenolic content

**FIGURE 1:**
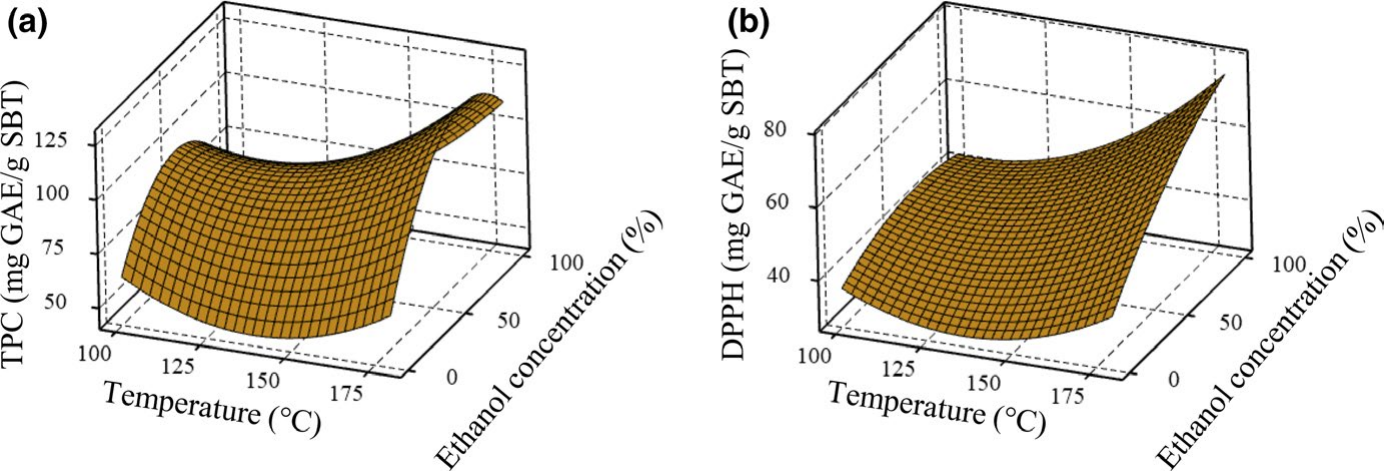
Response surface plots illustrating the effect of temperature and ethanol concentration on (a) the total phenolic content and (b) the DPPH antioxidant activity of spent black tea extract

A three‐dimensional surface graph was plotted of the results for the total phenolic content (TPC) of the SBT extracts (Figure 1a). The surface describes the variation of TPC as a function of the variables, over the range of values studied. This shows that raising the temperature up to a maximum of 180°C increased the phenolic content. This effect of temperature on TPC, confirmed in previous studies (Syahariza, Torkamani, Norziah, Mahmood, & Juliano, 2017; Vergara‐Salinas, Pérez‐Jiménez, Torres, Agosin, & Pérez‐Correa, 2012), was possibly caused by the enhanced solubility of the compounds and their increased mass transfer rate. However, ethanol concentration had a greater effect on phenolic content than temperature. The level of phenolics extraction did not increase linearly with increasing ethanol concentration but there was an optimum point after which the TPC decreased. This was confirmed by the most significant *p*‐value (*p* < .0001) of the quadratic term for ethanol concentration. It can also be seen that pure water (ethanol concentration 0%) even at a subcritical temperature was not suitable for extracting phenolic compounds but using water combined with an organic solvent can help to give better results. This phenomenon could have been caused by the creation of a reduced polar medium through using ethanol as the co‐solvent (Kwon & Chung, 2015; Mussatto, Ballesteros, Martins, & Teixeira, 2011). Nevertheless, polyphenols present in plant tissues have been found to be bound to proteins/polysaccharides by hydrogen and hydrophobic bonds. Therefore, this caused the yield using water extraction to be low, or possibly water alone cannot cleave hydrogen bonds (Miralai, Khan, & Islam, 2008). However, ethanol can precipitate polysaccharides and expel them from the solution (Xu et al., 2014). The interactive effect of two variables also showed a positive effect on the TPC. The polynomial equation obtained for the apparent phenolic content was as follows:

$$Y_{\text{TPC}}=266.1‐3.178\;X_{1}+0.716\;X_{2}+0.01143\;X_{1}^{2}‐0.01454\;X_{2}^{2}+0.00665\;X_{1}X_{2}$$

### 3.3 Effect variables on antioxidant activity

**FIGURE 2:**
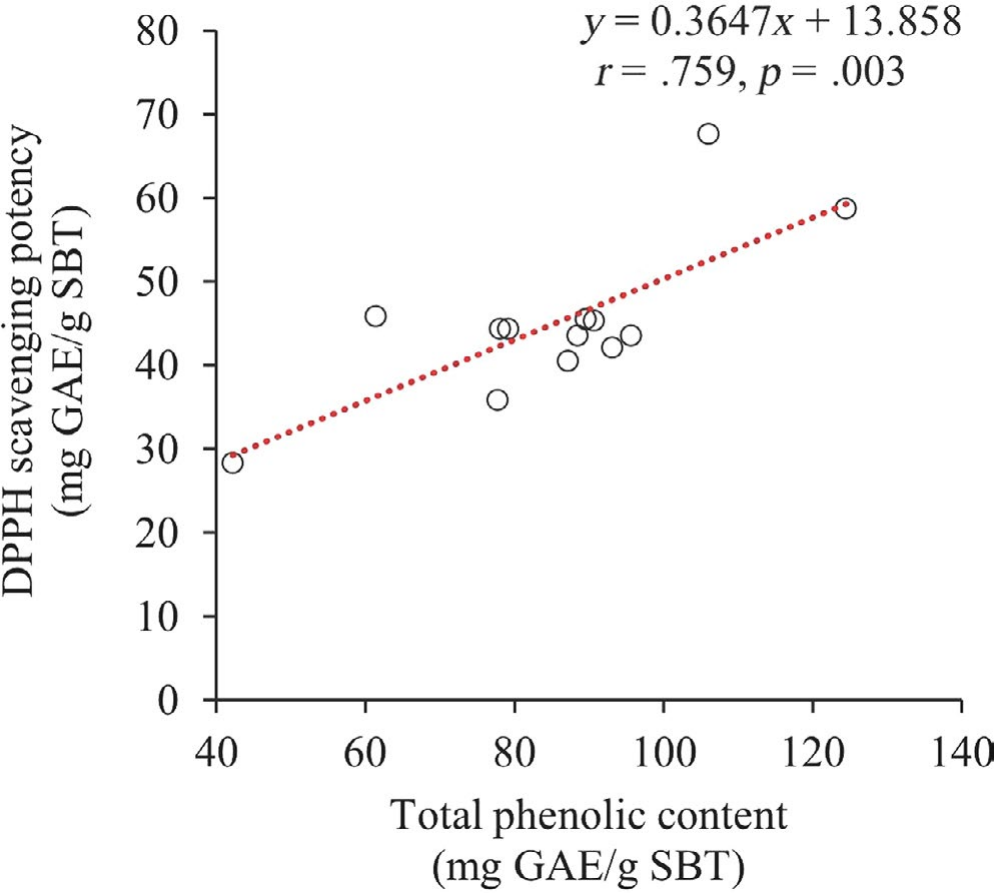
Correlation between DPPH scavenging activity and total phenolic content of SBT

The antioxidant activity was determined by the DPPH assay. Like TPC, DPPH scavenging capacity was plotted as a response surface diagram (Figure 1b). The DPPH values ranged between 36.78 and 75.61 mg GAE/g SBT with the lowest value observed for the conditions of 0% ethanol concentration and 100°C temperature. When comparing this plot with the surface plot of TPC, the patterns of the variation in responses with maximized regions were different. However, the values for TPC and DPPH scavenging capacity were significantly positively correlated (*r* = .759, *p* < .01) (Figure 2). The ANOVA revealed that the linear terms for temperature and ethanol concentration were most significantly affected by the responses of the antioxidant activity of the SBT extract. The quadratic term for temperature and the interactive effect of both variables demonstrated the positive effect on the responses as shown by the following equation:

$$Y_{\text{AO}}=141.2‐1.652\;X_{1}‐0.143\;X_{2}+0.0061X_{1}^{2}‐0.0017X_{2}^{2}+0.0037\;X_{1}X_{2}$$

### 3.4 Optimization of extraction process and experimental validation

**FIGURE 3:**
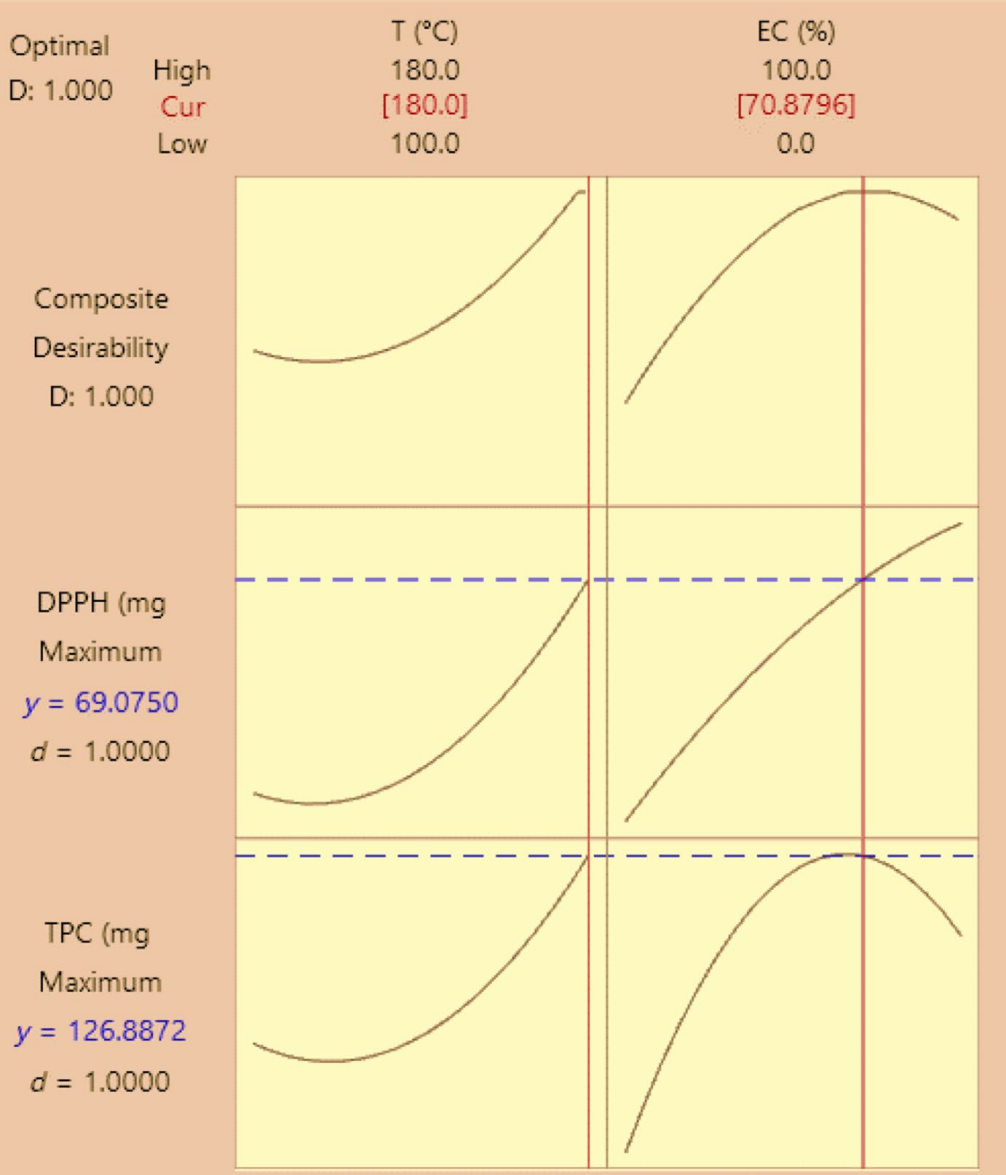
Optimization plot for the responses of total phenolic content (TPC, mg GAE/ g SBT) and antioxidant activity (DPPH, mg GAE/ g SBT)

**FIGURE 4:**
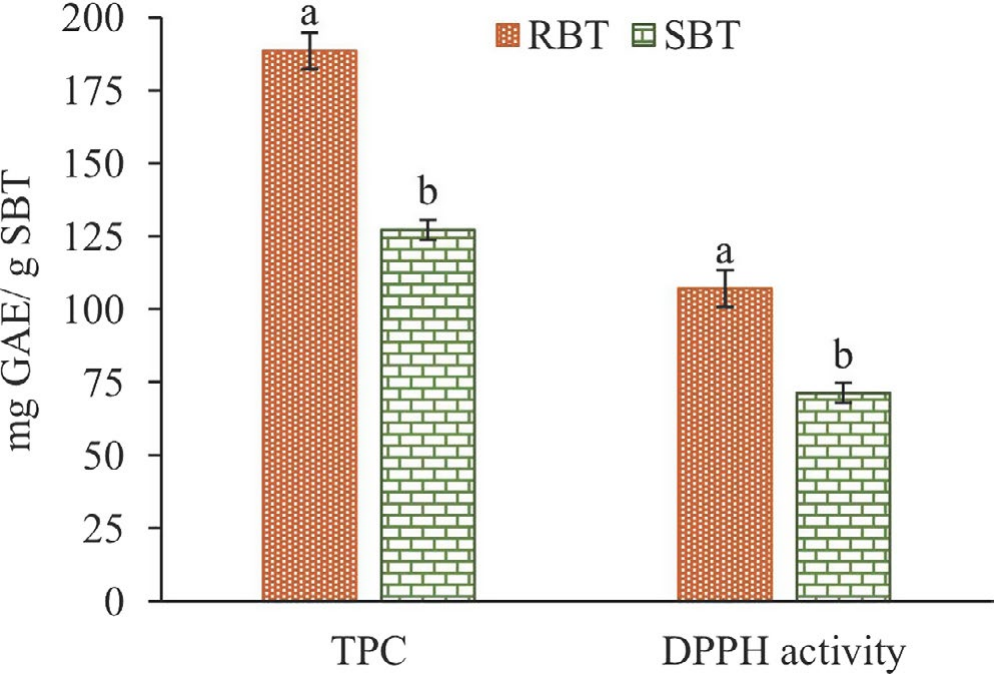
Comparison of the extract from raw black tea (RBT) and spent black tea (SBT) obtained under optimal conditions (180°C temperature and 71% ethanol concentration)

The SSE was optimized to yield an extract with a high content of phenolic compounds and a high antioxidant activity. A graphical optimization based on the effect of the two factors on the responses was conducted using the highest desirability level (Figure 3). This shows that under the optimal conditions (180°C temperature and 71% ethanol concentration), a TPC of 126.89 mg GAE/g SBT and DPPH activity of 69.08 mg GAE/g SBT can be obtained. These optimal conditions were used later for validation, and the results obtained for TPC (127.15 ± 1.67 mg GAE/g SBT) and DPPH activity (71.31 ± 3.40 mg GAE/g SBT) were very close to those predicted. Under the optimal conditions, the phenolic content and antioxidant activity of the raw and spent black tea were compared (Figure 4). The results showed that only 33% of the antioxidant phenolic compounds had been lost during tea brewing while the remaining 67% could be recovered using the optimized conditions for SSE. The optimal values of TPC from SSE in the present study were considerably higher than that (91.06 mg GAE/g SBT) reported in a previous study that used the maceration‐mediated liquid–liquid extraction of SBT (Mukhtar, Mushtaq, Akram, & Adnan, 2018).

### 3.5 Microencapsulation and microparticle characterization

#### 3.5.1 Particle size analyses

The particle size and size distribution are important characteristics of powder that can affect their storage and handling. To evaluate the effect of the type of wall material, the average particle diameter and size distribution were measured (Table 4). This revealed that the type of wall material significantly influenced the diameter of the particles (*p* < .05). The standard deviation values of the powder diameter measurements are related to the distribution of particles, with lower values indicating a more homogenous distribution. The microcapsules with SCN exhibited the lowest mean diameter (3.90 μm). The average particle size increased as the amount of pectin in the coating increased. Ahmadian et al. (2019) observed the same effect on the particle size with pectin. Particle size is related to the viscosity of the feed solution used for spray drying (Table 4). The solution with pectin possessed the highest viscosity and sodium caseinate the lowest. The particles with the mixture of wall materials exhibited the narrowest size distribution (1.45 μm) so were of a more uniform size than particles coated with the other two wall materials. This could have been caused by the heating of the pectin–sodium caseinate blend during its preparation, thus decreasing the polydispersity index by forming a more compact and dense particulate structure through re‐arranging the pectin molecules on the sodium caseinate surface (Liang & Luo, 2020).

**TABLE 4:** Effect of different wall materials on the viscosity of the feed solutions before spray drying, average particle diameter, and encapsulation efficiency of spray dried spent black tea powders

Sample	Viscosity of feed solution (mPa·s)	Mean particle diameter (μm)	Encapsulation efficiency (%)
PE	66.30^c^ ± 1.96	4.61^a^ ± 2.10	75.77^b^ ± 1.023
SCN	2.37^a^ ± 2.10	3.90^b^ ± 1.52	60.07^c^ ± 0.002
PE + SCN	19.35^b^ ± 2.30	4.50^ab^ ± 1.45	81.56^a^ ± 1.998

Note

Different letters in the same column indicate a statistically significant difference (*p* < .05) between mean values. Values represent the mean ± *SD* of three individual runs. Wall materials: PE, Pectin; SCN, Sodium caseinate; and PE + SCN, pectin and sodium caseinate.

#### 3.5.2 Encapsulation efficiency (EE%)

The encapsulation efficiency of SBT powder with coatings varied between 60.06% and 81.55%, thus confirming the successful entrapment of phenolic compounds within the wall materials (Table 4). The results also revealed that the type of coating agent used for encapsulation had an important role in retaining phenolic compounds in the carrier matrix (*p* < .05). The best result was achieved by the sample with SCN + PE (1:1 mass/mass). Pectin offers the advantageous as a protective carrier and its capability of interacting with hydrophobic molecules (Rehman et al., 2019). However, carbohydrates such as pectin mostly lack the interfacial functionality (Livney, 2010). Hence, it is better to combine polysaccharides with surface active biopolymer‐like caseinate to achieve successful encapsulation (Hogan, McNamee, O’Riordan, & O’Sullivan, 2001). Nevertheless, milk protein such as sodium caseinate can bind with polyphenols, especially with catechin, with this mechanism being promoted by a preheating treatment (Haratifar & Corredig, 2014; Shpigelman, Israeli, & Livney, 2010).

#### 3.5.3 Morphology

**FIGURE 5:**
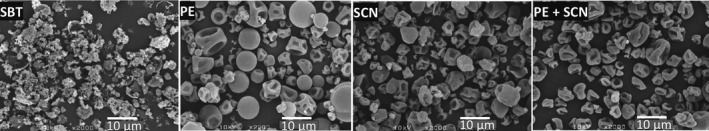
Micrographs spray dried powders loaded with spent black tea extract. SBT: phenolic powder spray dried with no encapsulating agents, PE: pectin, SCN, sodium caseinate, PE + SCN: pectin and sodium caseinate. Magnification 2000×

SEM was used to observe the microstructure of the encapsulated SBT extracts (Figure 5). Figure 5 shows that encapsulating SBT with wall materials effectively changed the external structure of the particles. The SBT powder with no coating materials exhibited an agglomerated and disordered structure while the encapsulated SBT powder formed apparently spherical particles with surface depressions. A similar observation was reported by Yinbin et al. (2018) when spray drying plum extract with different encapsulating agents. The powder coated with pectin appeared more spherical than that coated with other two wall materials investigated. However, the surface of most of the microparticles was wrinkled. This could have been affected by the lower inlet drying temperature (140°C). Microparticles produced at the higher drying temperatures exhibited a smoother surface with less shrinkage (Shamaei, Seiiedlou, Aghbashlo, Tsotsas, & Kharaghani, 2017; Yingngam et al., 2018). Particles with a coating of pectin and a combination of pectin and sodium caseinate exhibited fewer cracks and fissures which would have reduced the retention of polyphenols through contact with the external air and adverse heat conditions.

#### 3.5.4 Thermal stability

**FIGURE 6:**
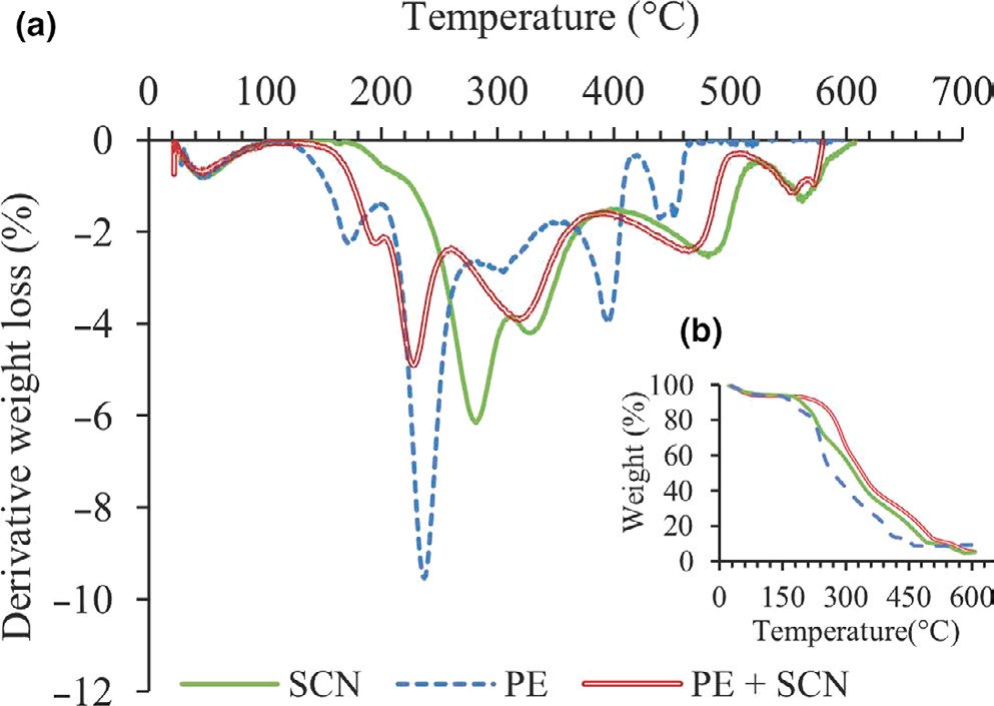
DTG (a) and TGA (b) thermographs of microencapsulated SBT powders using pectin (PE), sodium caseinate (SCN), and SCN + PE as wall materials

The thermogravimetric (TG) and derivative thermogravimetric (DTG) curves obtained from microparticles allow the visualization of their thermal degradation behavior (Figure 6a). All the tested samples exhibited multistage decomposition. The first stage of mass loss occurring between 30 and 100°C could have been caused by dehydration from the microencapsulated particles. The onset temperature for the second stage of decomposition of samples was observed in TGA curves at 120°C for SBT with pectin wall material, at 150°C for the combination of wall materials and at 210°C for the sample with sodium caseinate wall material. Thus, sodium caseinate as a wall material provided greater thermal stability than the other two materials. This was possibly because, with its flexible and disordered structure, it is less sensitive to changes in temperature (McClements, 2018). Figure 6a shows that the rate of weight loss during decomposition was lowest in the particles coated with a combination of wall materials possibly because of the enhanced electrostatic interaction between pectin and sodium caseinate (Chang et al., 2017). Pectin is an anionic polysaccharide that can interact with casein mainly through electrostatic, steric, or covalent interactions.

#### 3.5.5 Crystallinity

**FIGURE 7:**
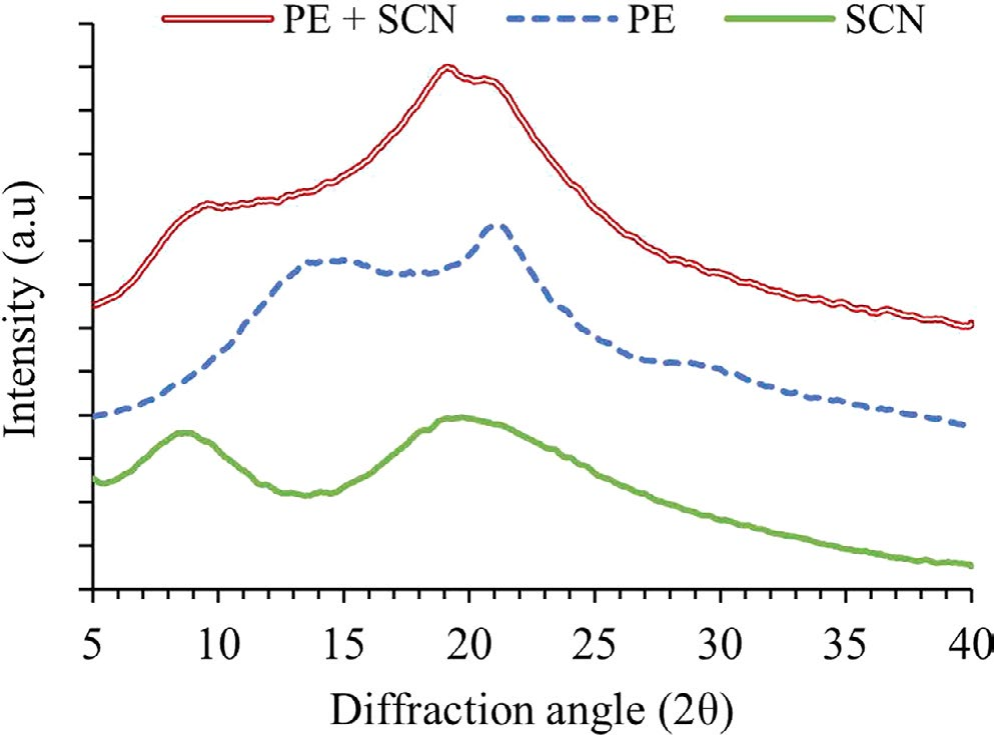
X‐ray diffraction pattern of microencapsulated SBT powders using pectin (PE), sodium caseinate (SCN), and SCN + PE as wall materials

The XRD patterns of the encapsulated SBT extracts coated with three polymer mixtures are shown in Figure 7. The results of XRD were evaluated after smoothing by using Match! Software (Crystal Impact, Bonn, Germany). Normally, a crystalline fraction diffracts X‐ray coherently according to Bragg′s law to give a sharp peak while an amorphous fraction diffracts incoherently to give a diffuse halo (Chung, 1999). Thus, in Figure 7, the XRD patterns with their wide bases indicate the amorphous structure all the obtained microcapsules. The degree of crystallinity of the microparticles coated with pectin, sodium caseinate, and the blend of pectin–caseinate was 5.75% ± 0.8%, 6.17% ± 0.4%, and 4.8% ± 0.9%, respectively. These results show that all coating treatments produced samples containing mostly an amorphous phase with the highest proportion (95.2%) in the particles coated with a mixture of wall materials. These results agreed with XRD results in previous studies on pectin (Hosseinnia, Khaledabad, & Almasi, 2017) and sodium caseinate (Pan, Zhong, & Baek, 2013). Forming of an amorphous structure enhances the rate of dissolution and solubility compared with a crystalline material thereby increasing the bioavailability of the active material (Kanaujia, Poovizhi, Ng, & Tan, 2015).

#### 3.5.6 Accelerated storage stability

**FIGURE 8:**
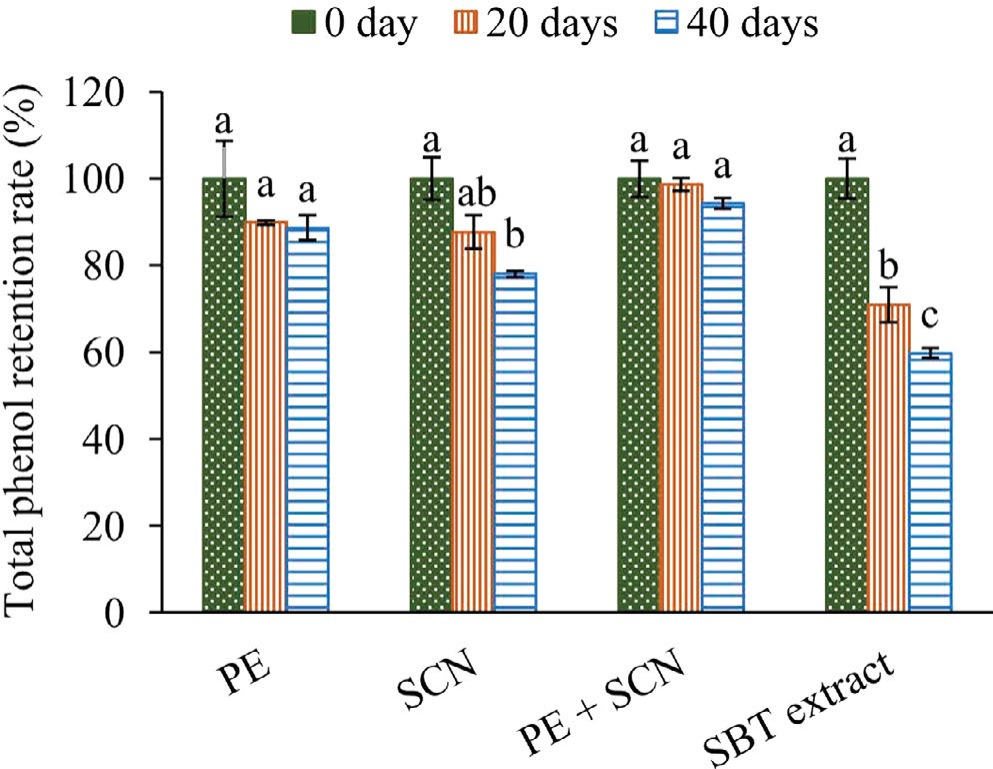
Changes in the phenolic content of SBT extract and microencapsulated powder with different wall materials. Values represent the mean (*n* = 3). Different letters in each coating material indicated a statistically significant difference (*p* < .05) between time points

The stability of the encapsulated SBT extract was evaluated (Figure 8). Irrespective of the type of wall material, the coatings preserve the core polyphenols than that of SBT extract without coatings. After 40 d of storage, the SBT extract with no coatings retained only 59.79% of its phenolic content, but the encapsulated powder contained 87.00% phenolic content on average for the three types of wall material. Similar results were reported by Zheng, Ding, Zhang, and Sun (2011) and Tsali and Goula (2018), who evaluated phenolic extracts and their microcapsules from by bayberry and grape pomace, respectively. This effective encapsulation from using a wall material enhances the shelf life of polyphenols in their extracted form by avoiding the damage caused by exposure to oxygen, high temperature, and humidity, etc., during storage.

Particles coated with the combination of wall materials provided a greater level of preservation of the core polyphenols compared with other two types. These results emphasize the effectiveness of blending pectin with sodium caseinate for use as a wall material as confirmed by the observations on morphology, encapsulation efficiency, and crystallinity.

## 4 CONCLUSIONS

Subcritical solvent extraction with ethanol as a co‐solvent was an efficient method for extracting phenolic compounds from spent black tea. The linear and combined interaction of temperature and ethanol concentration had a significant effect on the total phenolic content and the antioxidant activity of the SBT extract. The optimal extraction conditions were achieved at 180°C temperature and at an ethanol concentration of 71%. For microencapsulation, the greatest entrapment and preservation of antioxidant phenolic compounds were obtained by particles with a blend of pectin and caseinate blend as wall material. Using sodium caseinate was more appropriate for producing a more thermally stable microcapsules with the finest and smallest particles thus enhancing the handling of the powder. The proposed model based on a central composite inscribed design could be used for the subcritical solvent extraction of phenolic compounds in spent black tea, and the subsequent microencapsulation could enhance the stability of the polyphenols. Therefore, antioxidant phenolic compounds can be effectively recovered from spent black tea a food manufacturing waste product with subsequent microencapsulation turning it into valuable food ingredient.

## CONFLICT OF INTEREST

The authors declare that they have no conflict of interest.

## ETHICAL APPROVAL

Ethical approval is not required for this research.

## Data Availability

The research data are not shared.
